# Evaluation of radioactivity concentrations from the Fukushima nuclear accident in fish products and associated risk to fish consumers

**DOI:** 10.1093/rpd/nct239

**Published:** 2013-09-29

**Authors:** Jing Chen

**Affiliations:** Radiation Protection Bureau, Health Canada

Radioactive contamination of the Pacific Ocean following the Fukushima nuclear accident has raised public concerns about seafood safety^(1, 2)^. Many people are wondering whether fish products from the Pacific Ocean and Japan are safe to eat 2 y after the accident. There is also some concern about seafood caught locally, outside of Japan. Based on monitoring data reported in July 2013, radioactive caesium concentrations in fish products from Fukushima and adjacent prefectures are evaluated. Resulting radiation doses from annual consumption at average contamination levels and occasional fish meals at much higher levels of caesium are calculated. To put radiation doses from caesium intake in perspective, comparisons are made to doses from naturally occurring radioactive polonium commonly found in fish. Discussion and conclusions are given subsequently.

The Tokyo Electric Power Company has conducted routine radioactivity measurements (^134^Cs and ^137^Cs) of various marine fish and shellfish in the ocean area within a 20-km radius of the Fukushima Daiichi Nuclear Power Station (FDNPS). Based on their posted summary on 16 August 2013^(3)^, a total of 100 fish samples were collected from 7 to 23 July, offshore of the FDNPS and outside of its port area. The nuclide analysis report showed that ^134^Cs was detected in 64 samples with concentrations varying from 3.5 to 130 Bq kg^−1^. ^137^Cs was detected in 79 samples and the concentration varied from 3.6 to 260 Bq kg^−1^. On average, fish and shellfish caught within 20-km offshore of the FDNPS contain 12 Bq kg^−1^ of ^134^Cs and 26 Bq kg^−1^ of ^137^Cs.

The Japanese Fisheries Agency (JFA), in cooperation with the relevant prefectural governments and organisations, has conducted sampling and inspections of fishery products at the major fishing ports in Fukushima and adjacent prefectures on a weekly basis to examine the possible contamination of fishery products by radioactive materials released from the FDNPS. Based on press releases made by the JFA in July 2013^(4)^, a total of 1952 fishery products were analysed for caesium. Among them, ^134^Cs was found in 485 samples (25 %) and ^137^Cs was detected in 776 samples (40 %). The analyses were done by various institutions and the laboratory detection limits varied from <1 Bq kg^−1^ to ∼10 Bq kg^−1^. Among those samples with concentrations above detection limits, the reported ^134^Cs concentrations varied from 0.1 to 338 Bq kg^−1^ with an average of 11 Bq kg^−1^. The reported ^137^Cs concentrations varied from 0.1 to 699 Bq kg^−1^ and the mean of the reported values is 18 Bq kg^−1^. The highest concentrations (338 Bq kg^−1^ for ^134^Cs and 699 Bq kg^−1^ for ^137^Cs) were reported on 11 July from a sample of sea bass, offshore of Hitachi city. When averaged over all 1952 samples (with zero concentration for those below the detection limit), fish products from a broad ocean area in eastern Japan contain on average 3 Bq kg^−1^ of ^134^Cs and 7 Bq kg^−1^ of ^137^Cs.

Naturally occurring radionuclides exist in the environment in the air we breathe and in the water we drink. Not surprisingly, naturally occurring radionuclides can be found in varying concentrations in fishery products. A literature review conducted by Hosseini *et al*.^(5)^ revealed that the mean activity concentrations of natural occurring radionuclides in generic marine fish are 83 Bq kg^−1^ of ^40^K, followed by 30 Bq kg^−1^ of ^210^Po, 19 Bq kg^−1^ of ^14^C and 1.8 Bq kg^−1^ of ^228^Ra. Potassium is ingested in many foods that we eat and is a critically important element for proper functioning of the human body. Because the potassium content of the body is under strict homeostatic control in which the amount retained is actively regulated by the body, the ^40^K content in the human body is constant and not influenced by variations in environmental levels. Therefore, the effect of ^40^K intake from fish consumption will not be considered in the following discussion. For similar reasons, the effect of ^14^C will also be excluded in the subsequent dose assessment.

## ANNUAL FISH CONSUMPTION

The radiation dose resulting from fish consumption can vary widely depending on the concentrations of radioactive nuclides in the fish products. To assess radiation doses resulting from long-term fish consumption, such as annual fish intake, the concentration values averaged over broad ocean areas and fish products are used. This is because people normally get fish products from various sources during a year, and ocean fishes migrate significant distances during their lifecycles and in different seasons. In this study, radiation doses due to annual fish consumption are calculated with the average concentrations of 3 Bq kg^−1^ of ^134^Cs, 7 Bq kg^−1^ of ^137^Cs, 30 Bq kg^−1^ of ^210^Po and 1.8 Bq kg^−1^ of ^228^Ra.

Statistics of fish consumption fluctuate over the years, such as in Canada for example. Based on the statistics given by Fisheries and Oceans Canada^(6)^, averaged over the past 23 y (1988–2010), the Canadian consumption rate for fish products is 8.8 kg (edible weight) per person per year. This includes fresh and frozen sea fish, processed sea fish, total shellfish and freshwater fish. Fish consumption habit can vary significantly among individuals of a population, and differ significantly from a population to another. Therefore, the radiation dose is also calculated for 1 kg of fish intake. Since radiation doses are additive, one can easily find out his/her annual dose based on the amount of fish consumed.

Based on dose coefficients (DC) given in the International Commission on Radiological Protection Publication 119^(7)^, radiation doses resulting from consumptions of 1 and 8.8 kg fish products are given in Table 1 for children (5 y old) and adults. On average, a 5-y-old child may eat less fish than adults. However, without detailed age-specific statistics it is assumed that children consume the same amount of fish products as adults.

**Table 1.** Radiation doses due to intake of 1 and 8.8 kg of fish products from Pacific Ocean in eastern Japan.

	Concentration (Bq kg^−1^)	DC (nSv Bq^−1^)		µSv (from 1 kg)		µSv (from 8.8 kg)	
		Children	Adults	Children	Adults	Children	Adults
^134^Cs	3	13	19	0.039	0.057	0.34	0.50
^137^Cs	7	9.6	13	0.067	0.091	0.59	0.80
^210^Po	30	4400	1200	132	36	1162	317
^228^Ra	1.8	3400	690	6.1	1.2	54	11

One can see from Table 1, fish products from the contaminated Pacific Ocean in eastern Japan on average contain radioactive caesium, however, the concentrations are insignificant compared with the levels of naturally occurring radionuclides in fish. For an annual fish consumption of 8.8 kg, radioactive caesium would contribute a radiation dose of 1.3 µSv to adults and 0.9 µSv to children. These doses are only a small fraction of the radiation doses due to naturally occurring radionuclides normally found in fish, 0.4 % for adults and 0.08 % for children.

## OCCASIONAL FISH CONSUMPTION

When averaged over a wide range of fish products from broad ocean areas in eastern Japan, the contamination levels for radioactive caesium are very low. However, occasionally, individuals may have meals made from heavily contaminated fish. In this study, it is assumed a typical serving of fish for a meal would be 150 g. The highest contamination levels reported by TEPCO and JFA in July 2013 are used in the assessment even though these products would not be allowed to enter the marketplace as they exceed guideline levels issued by Japanese Government^(8)^. Radiation doses are then calculated for one fish meal of 150 g and several fish meals up to 1 kg, again for children (5 y old) and adults, respectively. Results are summarised in Table 2.

**Table 2.** Radiation doses due to intake of one fish meal (150 g) and several fish meals up to 1 kg from contaminated Pacific Ocean in Japan.

	Concentration (Bq kg^−1^)	DC (nSv Bq^−1^)		µSv (from 150 g)		µSv (from 1 kg)	
		Children	Adults	Children	Adults	Children	Adults
^134^Cs	3	13	19	0.0059	0.0086	0.039	0.057
	130	13	19	0.25	0.37	1.7	2.5
	338	13	19	0.66	0.96	4.4	6.4
^137^Cs	7	9.6	13	0.010	0.014	0.067	0.091
	260	9.6	13	0.37	0.51	2.5	3.4
	699	9.6	13	1.0	1.4	6.7	9.1
^210^Po	30	4400	1200	20	5.4	132	36
^228^Ra	1.8	3400	690	0.92	0.19	6.1	1.2

One can clearly see from Table 2, even if heavily contaminated fish are caught and consumed, the resulting radiation doses would still be much less than radiation doses from naturally occurring radionuclides commonly found in fish. At the highest concentrations reported in July 2013 (338 Bq kg^−1^ for ^134^Cs and 699 Bq kg^−1^ for ^137^Cs), one fish meal of 150 g can give a radiation dose of 1.7 µSv to children and 2.3 µSv to adults. The doses are 8 % and 42 % of the radiation doses from naturally occurring radionuclides commonly found in fish for children and adults, respectively.

## FISH CONSUMPTION UP TO A DOSE OF 0.1 mSv

Radioactive caesium (^134^Cs and ^137^Cs) are artificial radionuclide released from human sources, such as nuclear weapons tests or nuclear accidents. While ^134^Cs has a half-life of about 2 y, ^137^Cs has a half-life of 30 y. With such a long half-life, ^137^Cs can exist in the environment for an extended period after the accident. Caesium contaminated fish will thus be continuously caught in the Pacific Ocean in the years to come. For food safety, people tend to consider limiting the consumption of contaminated fish to an acceptable annual radiation dose in non-emergency conditions. For the purpose of radiation protection, the International Commission on Radiological Protection recommends an annual dose limit of 1 mSv to the public. In compliance with this public dose limit, an intervention level of 1 mSv is applied to different food groups independently in nuclear emergencies^(9, 10)^. However, for non-emergency conditions, a reference dose level of 0.1 mSv y^−1^ is normally proposed for a single type of food intake, such as the intake of drinking water^(11, 12)^ and thus fish consumption in the current consideration. In this way, the total dose from various possible types of intake or exposure pathways will likely not exceed the dose limit of 1 mSv y^−1^.

If individuals wish to limit their fish consumption within the reference dose level of 0.1 mSv y^−1^ as for normal living situations, the annual fish consumption can be calculated based on contamination levels, as given in Table 3. The estimated annual consumption in Table 3 are for total caesium, i.e. the sum of ^134^Cs and ^137^Cs during or shortly after radioactive releases and only ^137^Cs for years after releases have ceased. In the calculations, the dose coefficient of ^137^Cs is used. The dose coefficients of caesium are lower for children than for adults. The estimated annual consumption for adults should be conservative enough for the purpose of radiation protection of children.

**Table 3.** Annual consumption of contaminated fish with various caesium concentrations for a radiation dose of 0.1 mSv y^−1^ in non-emergency conditions.

Caesium (Bq kg^−1^)	DC (nSv Bq^−1^)	Annual consumption (kg)
10	13	769
50	13	154
100	13	77
500	13	15
1000	13	8
5000	13	2

On average, Canadians consume about 8 kg of fish per year. If the aim is to keep the additional radiation dose from fish consumption alone below 0.1 mSv as in non-emergency conditions, caesium concentration in fish products should be kept below 1000 Bq kg^−1^. Among 1952 fish samples reported by the JFA in July 2013, only one sample reached 1000 Bq kg^−1^ (338 Bq kg^−1^ of ^134^Cs and 699 Bq kg^−1^ of ^137^Cs). Although highly unlikely, if individuals happen to consume 8 kg of fish containing a total of 8000 Bq of caesium, the resulting radiation dose will be 0.1 mSv, which is only 5 % of the total background radiation dose (2 mSv y^−1^).

The majority of fish caught from Japanese coastal waters contain radioactive caesium much lower than 100 Bq kg^−1^, since the average is around 10 Bq kg^−1^. This means, practically, there should be no fish consumption limit, because people are not likely to eat several hundred kilograms of fish a year. If an individual does consume abundant quantities of fish per year, instead of radioactive caesium, naturally occurring radionuclides and other chemical contaminants commonly found in fish products could become a health concern. General advisories of fish consumption issued by governmental organisations should then be followed in those cases.

In summary, based on press releases given by the JFA in July 2013, about 2000 samples of fish products from Fukushima and adjacent prefectures were analyzed for radioactive caesium. 40 % of the samples have caesium levels above the laboratory detection limit, and 1.5 % of samples have levels above 100 Bq kg^−1^. Thus, the sampling and inspection showed that the majority of the fish products tested have non-detectable or low levels of caesium contamination. On average, fish products from Japan's coastal or offshore waters have 3 Bq kg^−1^ of ^134^Cs and 7 Bq kg^−1^ of ^137^Cs. For an annual fish consumption of 8.8 kg, radioactive caesium would contribute an annual radiation dose of 1.3 µSv to adults and 0.9 µSv to children. Those additional doses are only a small fraction of the radiation dose from background radiation.

In a worst case of consuming fish at the highest concentration (about 1000 Bq kg^−1^ of caesium) reported in July 2013, one normal fish meal of 150 g can give radiation doses of 1.7 µSv to children and 2.3 µSv to adults. The doses are 8 and 42 % of radiation doses from naturally occurring radionuclides commonly found in fish for children and adults, respectively.

The results presented here are for fish products from contaminated waters around Japan. This study provides the upper boundary estimates if individuals only consume fish products from Fukushima and adjacent prefectures in Japan. Even in those worst cases, there is no real health concern based on monitoring data reported to date, since caesium levels in 98.5 % of Japanese fish products are well below 100 Bq kg^−1^, and the other 1.5 % of fish products having total caesium >100 Bq kg^−1^ are not allowed to enter the market according to the Japanese regulatory authorities^(13)^.

The longer lived caesium isotopes (2 y half-life for ^134^Cs and 30 y half-life for ^137^Cs) could be transported over long distances by ocean currents, mainly eastwards by the Kuroshio current system. However, the great quantity of water in the Pacific Ocean will rapidly disperse and dilute these radioactive materials. Testing of seawater 30 km off the coast of Japan has shown that the concentrations of radionuclides have dropped rapidly to very low levels and are of no public health concern^(2)^. Some of the contaminated fish in Japanese coastal waters such as tuna and salmon can migrate to other ocean areas. However, migratory fish normally spend most of their transoceanic migration period outside of Japan's coastal or offshore water. In addition, radioactive caesium declines when fish leave contaminated waters as caesium is not bound in the body and will gradually be excreted from the fish. The biological half-life of caesium in sea fish is typically between 5 and 100 d ^(2, 14)^. Therefore, the caesium level in fish caught outside of Japan is most likely undetectable. If any is detected in those seafood, the contamination will be significantly below any public health concern. Fish products outside of Japan are not of radiological health concern, even for individuals with high seafood consumption.

For radioactive caesium emitting beta and gamma radiation, doses resulting from a unit intake are lower in children than in adults. The fish consumption limits estimated for adults in regard of caesium contamination are valid for the purpose of radiation protection to children as well. However, for naturally occurring radioactive polonium (emitting alpha particles) commonly found in fish, the resulting dose from unit intake is about four times higher to a child of 5 y old than to an adult. In cases of insignificant contamination of caesium or no radioactive contamination at all, fish consumers should also check advisories issued by local governments for the health concerns from naturally occurring radionuclides and other chemical contaminants, especially for the protection of children.
